# Non-genetic factors affecting pre-weaning growth and morphometric traits in Assam Hill goat

**DOI:** 10.14202/vetworld.2019.1327-1331

**Published:** 2019-08-25

**Authors:** L. Sarma, N. Nahardeka, R. N. Goswami, A. Aziz, G. Zaman, A. Das, F. Akhtar

**Affiliations:** 1 Department of Animal Genetics and Breeding, College of Veterinary Sciences, Assam Agricultural University, Guwahati, Assam, India; 2 Goat Research Station, Assam Agricultural University, Burnihat -793101, Assam, India

**Keywords:** Assam Hill goat, genetic parameters, morphometric traits, non-genetic factors, pre-weaning growth

## Abstract

**Aim:**

This study aimed to determine the genetic and non-genetic factors affecting pre-weaning body weight (BW) and morphometry in Assam Hill goat along with the genetic parameters.

**Materials and Methods:**

The detailed information in respect of BW and body measurements of 960 animals at birth and 3 months of age belonging to three different populations of Assam Hill goat maintained at field units, namely, Batabari, Nahira, and Tetelia under “All India Coordinated Research Project on Goat Improvement” were utilized in the present study. The data were analyzed using least squares technique.

**Results:**

The least squares means for BW, height at withers (HW), heart girth (HG), and body length (BL) were 1.166±0.008 kg, 26.198±0.070 cm, 26.695±0.096 cm, and 29.482±0.119 cm at birth and 4.590±0.083 kg, 36.850±0.105 cm, 40.741±0.115 cm, and 39.703±0.108 cm at 3 months of age, respectively. Location had a significant effect on BW, HW, and BL at both birth and 3 months and on HG at 3 months of age. Season of birth exerted significant effect only on BL at birth, whereas the significant effect of sex was observed on HG and BL at 3 months of age. The heritability estimates for BW and body measurements were moderate indicating the scope of selection. The phenotypic and genetic correlations among BWs and body measurements at birth and 3 months of age were positive in direction and high in magnitude.

**Conclusion:**

On the basis of the present findings, it could be concluded that the weaning weight of kids can be considered for the selection of parent stock to increase productivity and eventually the economic efficiency. Further, animals with higher body measurements at initial phases of growth will perform better with respect to even BW at later stages of growth.

## Introduction

Goat substantially contributes to the rural economy and provides a livelihood to the poor sections of the society. India possesses the second largest goat population in the world with 135.17 million goats (26.40% of the country’s total livestock) which corresponds to 15.68% of total goat population in the world [1]. The goat population of Assam is 6.169 million, contributing 4.56% of total goat population of India [1]. The Assam Hill goat is a newly registered breed distributed throughout the state of Assam possessing good quality meat and high adaptability characteristics to local high humid agro-climatic condition. Almost the entire population of Northeastern region of India is non-vegetarian and chevon is the meat of choice having no religious taboo. The chevon production in Assam in the year 2012-2013 was 11,000 tonnes which were 20% of the total meat produced in the state [1]. The gap between demand and production of meat could be bridged by augmenting the growth performance and reproductive efficiency of the animals.

Birth weight is an important parameter in meat-producing animals due to its strong correlation with growth rate and adult size as well as with the viability of newborn animals, while weaning weight has a high relative economic importance to the farmers [2]. Evaluation of early growth and morphometric traits indicates the growth trends which enable more accurate assessment of growth potentials; better understanding of the impact of genetic and environmental factors on growth; more accurate prediction of mature growth and linear body values before attainment of maturity; and early selection, reduction of generation interval, and rapid genetic progress [3]. In meat-producing animals, external body measurements could be a reliable indicator of its future performance with respect to live body weights (BWs), if and only if a correlation has been identified among these traits of interest [4]. To formulate efficient breeding plans and selection strategies, knowledge of genetic parameters (heritability) among various traits is essential [5,6].

This study aimed to determine the genetic and non-genetic factors affecting pre-weaning BWs and morphometry in Assam Hill goat along with the genetic parameters.

## Materials and Methods

### Ethical approval

The present work has been approved by the Institutional Animal Ethics Committee of Assam Agricultural University, vide Approval No. 770/ac/CPCSEA/FVSc/AAU/IAEC/15-16/285 dated 10.04. 2015.

### Location and data collection

The animals used in this study were reared by the farmers belonging to the field units under “All India Coordinated Research Project on Goat Improvement,” Goat Research Station, Assam Agricultural University, Burnihat. The location lies between 90.48° and 92.22° (East) longitude and 20.09° and 26.95° (North) latitude. The data pertaining to 960 animals were utilized in the present investigation. Measurements of four morphological characters, namely, BW, height at withers (HW), heart girth (HG), and body length (BL) were recorded at birth and 3 months of age according to measurement regions [7]. Measuring tapes were used to record the length and HG in centimeter, while the heights were measured using graduated stick. The BWs were measured using spring balance of 50 kg capacity in the morning hours. The birth weights of the kids were recorded within 12 h of birth. The data were classified into three locations, namely, Batabari (L_1_), Nahira (L_2_), and Tetelia (L_3_); four seasons of birth, namely, pre-monsoon, i.e., March-May (S_1_), monsoon i.e., June-September (S_2_), post-monsoon, i.e., November-December (S_3_), and winter, i.e., December-February (S_4_); and two sexes as male and female.

### Management practices

The goat sheds were made of locally available materials, i.e., bamboo, wood, thatches, etc. The slotted floors of the houses were raised by about 3 ft. The walls of the shed were made of bamboo and covered up to three-fourth of the total height of the wall and rest of the portions were kept open for ventilation. The animals were kept under semi-intensive management system. Selective breeding was practiced where the breeding does were mated with superior bucks supplied under the project. All the male kids except those kept for breeding purpose were castrated before 3 months of age. The animals were immunized regularly against enterotoxemia, peste des petits ruminants, and goat pox. Regular deworming was practiced in all the field units under study.

### Statistical methods

The data were analyzed using least squares means (LSM) as described by Harvey [8]. The statistical model used to analyze the data was Y_ijko_ = µ+L_i_+M_j_+S_k_+e_ijko_, where Y_ijko_ is the value of the o^th^ animal in the (ijk)^th^ subclass, µ is the overall population mean, L_i_ is the effect of i^th^ location (i = 1, 2, 3), M_j_ is the effect of j^th^ season of birth (j = 1, 2, 3, 4), S_k_ is the effect of the sex of the kid (k = 1, 2), and e_ijko_ is the random error associated with Y_ijko_ assumed to be NID (0, σ^2^_e_). Duncan’s multiple range test (DMRT) was done to make pairwise comparison among the means wherever significant differences exist.

The estimates of heritability (h^2^), phenotypic (r_P_), and genetic (r_G_) correlations were calculated from the sire component of variance and covariance. The statistical model used to estimate the genetic parameters was Y_ij_ = µ+S_i_+e_ij_, where Y_ij_ is the observed value of j^th^ progeny on i^th^ sire, µ is the overall population mean, S_i_ is the effect of i^th^ sire common to all its progeny (i = 1,2,3…), and e_ij_ is the random error component assumed to be NID (0, σ^2^_e_). Standard errors (SEs) of heritability, phenotypic, and genetic correlation were estimated using the standard statistical methods.

## Results and Discussion

The estimates of LSM and SE for BW, HW, HG, and BL at birth and 3 months of age along with the results of DMRT are presented in Table-1, and least squares analyses of variance showing the effect of different factors on the traits are presented in Table-2.

**Table 1:** Least squares means and standard errors for body weight (kg), height at withers (cm), heart girth (cm), and body length (cm) along with the results of Duncan’s multiple range test.

Effects	n	LSM±SE							

		BW		HW		HG		BL	
			
		Birth	3 months	Birth	3 months	Birth	3 months	Birth	3 months
μ	960	1.166±0.008	4.590±0.083	26.198±0.070	36.850±0.105	26.695±0.096	40.741±0.115	29.482±0.119	39.703±0.108
Location									
L1	212	1.181±0.015^a^	4.354±0.165^a^	25.982±0.140^a^	36.124±0.210^a^	26.682±0.192	39.859±0.280^a^	29.552±0.237^ab^	38.507±0.216^a^
L2	335	1.200±0.012^a^	4.541±0.134^ab^	25.992±0.113^a^	36.837±0.170^b^	26.560±0.155	39.347±0.175^b^	29.901±0.191^a^	40.424±0.175^b^
L3	413	1.120±0.011^b^	4.875±0.121b	26.619±0.102^b^	37.590±0.154^c^	26.843±0.140	43.017±0.298^c^	28.992±0.173^b^	40.177±0.158^b^
Season of birth									
S1	254	1.175±0.014	4.610±0.152	26.157±0.128	36.939±0.193	26.686±0.176	40.421±0.299	29.782±0.217^ab^	39.488±0.198
S2	280	1.160±0.014	4.446±0.148	26.425±0.125	36.873±0.188	26.673±0.172	41.107±0.230	29.626±0.211^a^	39.966±0.192
S3	150	1.155±0.018	4.889±0.196	26.118±0.166	36.563±0.250	26.664±0.228	40.562±0.331	30.014±0.281^b^	39.664±0.257
S4	276	1.175±0.013	4.416±0.146	26.091±0.123	37.025±0.186	26.756±0.170	40.874±0.263	28.504±0.209^c^	39.692±0.191
Sex									
Male	513	1.220±0.010	4.642±0.110	26.213±0.093	36.855±0.139	26.730±0.128	42.849±0.234^a^	29.658±0.157	40.104±0.143^a^
Female	447	1.114±0.011	4.538±0.117	26.182±0.099	36.845±0.149	26.669±0.136	38.633±0.181^b^	29.605±0.168	39.302±0.153^b^

Means of a subclass with at least one superscript in common do not differ significantly from each other (p<0.05). n=Number of observation, LSM=Least squares mean, SE=Standard error

**Table 2:** Least squares analyses of variance for body weight, height at withers, heart girth, and body length.

Sources of variation	df	MS							

		BW		HW		HG		BL	
			
		Birth	3 months	Birth	3 months	Birth	3 months	Birth	3 months
Location	2	0.647*	21.309*	14.357*	155.234**	7.300	4556.688**	36.570*	266.945**
Season of birth	3	0.022	8.683	6.124	7.217	0.431	4417.850	31.397*	10.212
Sex	1	0.032	2.609	0.223	0.024	0.618	6549.005**	14.607	38.126*
Error	953	0.157	5.734	4.102	9.300	7.757	986.296	11.759	9.801

* p<0.05,

** p<0.01. BW=Body weight, HW=Height at withers, HG=Heart girth, BL=Body length

### BW

The overall LSM±SE for birth weight were found to be 1.166±0.008 kg in the present study (Table-1) which is in conformity with the finding of Otuma and Onu [9] in Nigerian crossbred goats; while, the reports of Mia *et al*. [10] in Black Bengal goat support the mean value for weaning weight in the present study which was found to be 4.590±0.083 kg (Table-1). However, the present finding is higher than the finding of Miah *et al*. [11] for birth weight and the finding of Singh and Singh [12] for weaning weight in Black Bengal goats. In contrast, Syahirah *et al*. [13] reported higher values for both the traits in Boer kids.

### Effect of location

Location had a significant effect on both birth and weaning weight (Table-2). Kids born at Nahira unit exhibited higher birth weights as compared to Batabari and Tetelia unit. This might be due to better management practices adopted by the farmers of Nahira. Weaning weight of kids of Tetelia unit significantly differed from the kids of Batabari. This could be justified by proper postnatal care and management by the farmers of Tetelia unit along with availability of mother’s milk to the unweaned kids.

### Effect of season of birth

Non-significant effect of season of birth was observed on both the traits (Table-2), which is in agreement with the observations of Faruque *et al*. [14] in Black Bengal goats for birth weight and Bedhane *et al*. [15] in Arsi-Bale goats for weaning weight. However, a significant effect of season of birth on both the traits was reported by Mahal *et al*. [16] in Black Bengal goats.

### Effect of sex

Analyses of variance revealed that birth and weaning weight were not significantly affected by sex of kids though the weights of male kids were higher than the females (Table-2). Similar observations were obtained by Mia *et al*. [10] in Black Bengal goats. However, a significant effect of sex of kid was reported by Bedhane *et al*. [15] in Arsi-Bale goats for both the traits. The increase in BW of male goats might be attributed to the early activation of male gonads than the females. Male sex hormone testosterone responsible for the anabolic effect could have caused the faster growth in male animals.

### HW, HG, and BL

The overall LSM±SE for HW, HG, and BL were 26.198±0.070 cm, 26.695±0.096 cm, and 29.482±0.119 cm at birth and 36.850±0.105 cm, 40.741±0.115 cm, and 39.703±0.108 cm at 3 months of age, respectively (Table-1). These findings are in accordance with the findings of Pan *et al*. [17] in Black Bengal goats. However, much higher values were recorded in Sirohi goats for all the traits by Dudhe *et al*. [18].

### Effect of location, season of birth, and sex of kids on morphometric traits

Location affected HW and BL significantly at both the age groups, but HG at weaning age only (Table-2).

Non-significant effect of season of birth on HW and HG at both age groups was observed in the present investigation (Table-2). However, a significant effect on HG was observed by Dudhe *et al*. [18] in Sirohi goats. On the other hand, there was a significant effect of season of birth on BL at birth in the present investigation (Table-2). The mean BL was comparatively higher in goats born during post-monsoon season. The result is in accordance with the finding of Dudhe *et al*. [18] in Sirohi goats.

Although differences in HW at birth and weaning due to sex were not significant, the values of the LSM for male animals were higher than those for the females (Table-2). On the contrary, sex of kids exerted significant effects on HG and BL at weaning in the present study (Table-2) which is in the flow of the reports obtained by Dudhe *et al*. [18] in Sirohi goats. The values of HG were found to be higher in male goats than those of females. However, a non-significant effect was observed by Kharkar *et al*. [19] in Berari goats.

### Genetic parameters

The estimates of genetic parameters, namely, heritability (h^2^), phenotypic correlation (r_P_), and genetic correlation (r_G_) along with the SE among BWs and body measurements at birth and 3 months of age are presented in Tables-3 and 4.

**Table 3:** Estimates of heritability (h^2^), phenotypic (r_P_), and genetic (r_G_) correlation between birth and weaning weight.

r_G_	r_P_	

	Birth	3 months
Birth	0.2611±0.117	0.6455±0.019
3 months	0.4231±0.256	0.1169±0.067

N.B. The elements of the diagonal are the estimates of heritability, above the diagonal is the phenotypic correlation, and below the diagonal is the genetic correlation

**Table 4:** Estimates of heritability (h^2^), phenotypic (r_P_), and genetic (r_G_) correlation between body weight and body measurements at birth and 3 months of age.

r_G_	r_P_			

	BW	HW	HG	BL
Birth				
BW	0.2611±0.117	0.4492±0.032	0.4306±0.026	0.4357±0.032
HW	0.4430±0.302	0.1244±0.070	0.6334±0.017	0.7620±0.032
HG	0.5188±0.203	0.2173±0.341	0.2444±0.112	0.4772±0.032
BL	0.4051±0.270	0.4821±0.351	0.3588±0.297	0.2750±0.122
3 months				
BW	0.1169±0.067	0.6682±0.032	0.4991±0.032	0.4847±0.032
HW	0.4247±0.336	0.4406±0.174	0.5640±0.032	0.6835±0.032
HG	0.4426±0.364	0.3644±0.278	0.1567±0.081	0.5001±0.031
BL	0.3577±0.335	0.4381±0.291	0.6280±0.338	0.2643±0.118

N.B. The elements of the diagonal are the estimates of heritability, above the diagonal is the phenotypic correlations, and below the diagonal is the genetic correlations. BW=Body weight, HW=Height at withers, HG=Heart girth, BL=Body length

### Heritability

The heritability estimates for birth and weaning weight under study were moderate (Table-3), which are in the line of the estimates of Kenneth *et al*. [20] and Kuthu *et al*. [5] in meat goats of the USA and Teddy goats, respectively. Moderate estimates indicated the scope for improvement of the traits through selection. However, lower estimates of heritability were obtained by Faruque *et al*. [14] in Black Bengal goats for birth weight and Bedhane *et al*. [15] in Arsi-Bale goats for weaning weight. On the contrary, higher values than the present estimates were reported by Mia *et al*. [10] in Black Bengal goats for both the traits.

Similarly, moderate estimates of heritability were obtained for HW, HG, and BL at birth and weaning in the present study (Table-4) which also indicated scope of improvement by selection. However, Dudhe *et al*. [18] in Sirohi goats reported higher estimates of heritability for morphometric traits as compared to the present estimates.

### Phenotypic and genetic correlation

The phenotypic and genetic correlations among pre-weaning BWs were found to be positive and high at both the age groups (Table-3). It has considerable significance in the selection program for BW since BW at one of these early ages is a good indication of BW at 12 months of age. These high correlations are of economic importance and suggest that the genetic factors are largely similar for these early and 12 months of age. These findings are in conformity with the findings of Mia *et al*. [10] in Black Bengal goats.

The phenotypic and genetic correlations among BWs and various body measurements were found to be positive and high at both the age groups (Table-4). This indicated that the linear body measurements could reliably play a predictive role for BW at any given age. BW appeared to be a function of these body measurements. This indicated the possibility of improving BW as a correlated response by exerting selection pressure on the body measurements. Dudhe *et al*. [18] in Sirohi goats reported positive and high phenotypic and genetic correlation among body measurements as obtained in the present study.

## Conclusion

Weaning weight of kids can be considered for the selection of parent stock to increase productivity and eventually the economic efficiency. These results, therefore, provide an important perspective on the selection objectives of Assam Hill goats by considering different environmental factors. Since BW and body measurements are important parameters to describe growth, so selective breeding of Assam Hill goat based on these parameters needs to be given more emphasis for greater productivity.

## Authors’ Contributions

LS and NN designed the experiment and conducted the study; RNG, AA, and GZ analyzed and interpreted the data; and AD and FA revised the manuscript. All authors read and approved the final manuscript.
